# Impact of Using Corticosteroid Prophylaxis to Prevent Tumor Flare Reactions During ^177^Lu-DOTATATE Treatment in Patients with Neuroendocrine Tumors

**DOI:** 10.3390/cancers17213472

**Published:** 2025-10-29

**Authors:** Amanda S. Cass, Emily Skotte, Margaret C. Wheless, Shannon Stockton, Robert A. Ramirez

**Affiliations:** 1 Vanderbilt Ingram Cancer Center, Nashville, TN 37232, USA; amanda.s.cass@vumc.org (A.S.C.); emily.skotte@vumc.org (E.S.); shannon.stockton@flcancer.com (S.S.); 2 Department of Medicine, Division of Hematology/Oncology, Vanderbilt University Medical Center, Nashville, TN 37232, USA; margaret.wheless@vumc.org

**Keywords:** corticosteroids, ^177^Lu-DOTATATE, neuroendocrine tumor, tumor flare

## Abstract

**Simple Summary:**

^177^Lu-DOTATATE has changed the treatment landscape for patients with gastroenteropancreatic neuroendocrine tumors. As with all therapies, side effects are inevitable. For patients with a high burden of disease, treatment with ^177^Lu-DOTATATE can cause a tumor flare reaction, causing significant pain or other complications. There have been some reports on the use of corticosteroids to prevent this adverse effect. We aimed to see if we could achieve success at our institution when using corticosteroids to prevent a tumor flare reaction. We identified forty-six patients who receive corticosteroids in this setting. Twenty-eight percent of patients still had a tumor flare reaction with corticosteroid prophylaxis, and this statistic is similar to previously reported numbers. Twenty-eight percent of patients also experienced adverse effects from the corticosteroids used in this setting. Further studies are needed to examine the risks versus the benefits of corticosteroid use, so as to prevent tumor flare reactions in patients treated with ^177^Lu-DOTATATE with a high disease burden.

**Abstract:**

Background/Objectives: Since ^177^Lu-DOTATATE was approved for patients with somatostatin receptor (SSTR)-positive gastroenteropancreatic neuroendocrine tumors (NETs), tumor flare reactions including increased pain and small bowel obstruction (SBO) have been reported. Retrospective reviews report some success in using corticosteroids for treatment and prophylaxis of tumor flare reactions from ^177^Lu-DOTATATE. Given that corticosteroids are used in practice to help prevent tumor flare reactions based on limited evidence, we aimed to assess if this practice was efficacious in our patient population. Methods: In this retrospective and single-institution study, we identified adult patients with NETs who were treated with ^177^Lu-DOTATATE between 1 October 2019 and 31 December 2024; these patients received corticosteroids as prophylaxis for flare reactions due to high burden of disease, significant peritoneal or mesenteric disease, or disease involvement of critical structures as determined by the treating provider. Variables including demographics, diagnosis, treatment history, steroid dosing, and outcomes were collected within a RedCAP database. Results: Forty-six patients were identified as having received corticosteroid prophylaxis to prevent a tumor flare reaction due to ^177^Lu-DOTATATE. Patients had a median age of 66, and 50% were female. The primary disease site was the small intestine (72%) followed by the pancreas (9%). The majority of patients had World Health Organization (WHO) grade 1 (41%) or WHO grade 2 (35%) diseases. Most patients (83%) received corticosteroids prior to the initiation of ^177^Lu-DOTATATE, while 17% of patients received corticosteroids due to having a previous tumor flare after ^177^Lu-DOTATATE administration. Despite corticosteroid prophylaxis, 28% of patients still experienced a tumor flare event, with three patients experiencing multiple tumor flare events. Small bowel obstructions occurred in 7% of patients and increased abdominal pain in 22% of patients. Adverse events (AEs) due to corticosteroids occurred in 28% of patients. Conclusions: Short-course corticosteroid prophylaxis to prevent tumor flare reactions in high-risk patients with neuroendocrine tumors treated with ^177^Lu-DOTATATE did not appear to decrease the incidence of tumor flare reactions compared to previously reported numbers. Randomized, placebo-controlled trials looking at the use of corticosteroids to prevent tumor flare reactions in patients treated with ^177^Lu-DOTATATE are needed to fully elucidate the safety and efficacy of corticosteroids used in this setting and to determine the impact on treatment outcomes.

## 1. Introduction

Neroendocrine tumors (NETs) are rare tumors that can occur in almost any organ but are most commonly found in the gastrointestinal tract and can be hormone secreting or non-functional [1]. Although rare, the incidence of neuroencrine tumors continues to rise in the United States and on a global scale, particularly in females [1,2]. There are several different systemic treatment options for NETs, including somatostatin analogs (lanreotide, octreotide, and pasereotide), tyrosine kinase inhibitors (TKIs) such as sunitinib and cabozantinib, the mammalian target of rapamycin (mTOR) inhibitor everolimus, chemotherapy, and peptide receptor radionuclide therapy (PRRT) [3,4,5]. Since the United States Food and Drug Administration (FDA)’s approval of the PRRT medication ^177^Lu-DOTATATE, the treatment landscape has changed for patients with well-differentiated neuroendocrine tumors (NETs).

PRRT is a targeted form of radiotherapy comprising a peptide that binds to a specific receptor on a tumor cell combined with a radionuclide by a chelator [6,7]. Upon the binding of the receptors on tumor cell surfaces, the peptide–radionuclide complex is internalized, and radioactive therapy is delivered intracellularly [6,7]. ^177^Lu-DOTATATE comprises the radionuclide lutetium-177, the chelator DOTA, and the targeting peptide octreotate which targets somatostatin receptors (SSTRs) found on the cell surfaces of NETs [8,9]. Lutetium-177 is a β particle emitter which penetrates sufficiently to kill the targeted tumor cell while causing minimal damage to the surrounding tissue and organs [8,9]. PRRT can be logistically challenging for institutions requiring specialty centers to ensure its safe and effective management [10].

Approval of ^177^Lu-DOTATATE was based on the phase 3 NETTER-1 trial that compared ^177^Lu-DOTATATE plus octreotide LAR (long acting repeatable) 30 mg versus octreotide LAR 60 mg in patients with well-differentiated, metastatic somatostatin receptor (SSTR)-positive, grade 1 or 2 midgut neuroendocrine tumors who had progressed with first line therapy. The primary endpoint was progression-free survival (PFS) [11]. At data cutoff, the PFS at month 20 was 65.2% in the ^177^Lu-DOTATATE group compared to 10.8% in the octreotide-LAR-alone group [11]. Median PFS had not been reached in the ^177^Lu-DOTATATE group compared to the control group’s achievement of PFS at 8.4 months (*p* < 0.001) [11]. Additionally, the ^177^Lu-DOTATATE group had a response rate of 18% compared to 3% in the octreotide-LAR-alone group (*p* < 0.001) [11]. At the five-year final data analysis, overall survival was not statistically significant, with a median overall survival of 48.0 months in the ^177^Lu-DOTATATE group compared to 36.3 months in the control group [12]. However, the 11.7-month difference in median overall survival is debatably clinically significant and likely a result of the crossover design in the trial.

The NETTER-2 trial was a phase 3 trial that looked at ^177^Lu-DOTATATE plus octreotide LAR 20 mg compared to octreotide LAR 60 mg in patients with newly diagnosed, higher grade 2 (Ki67 ≥ 10 and ≤20%) and well-differentiated grade 3 (Ki-67 > 20% and ≤55%) SSRT-positive advanced gastroenteropancreatic NETs [13]. Median PFS was 22.8 months in the ^177^Lu-DOTATATE group compared to 8.5 months in the control group (*p* < 0.0001) [13]. The overall response rate was also significantly higher in the ^177^Lu-DOTATATE group at 43% compared to 9.3% in the octreotide-60 mg-LAR-alone group (*p* < 0.001) [13].

Although we do not see an overall survival benefit with ^177^Lu-DOTATATE in the NETTER-1 and NETTER-2 trials, the prolonged disease control in addition to clinical improvements in terms of symptom control cannot be underscored. In the NETTER-1 trial, patients completed quality of life (QoL) questionnaires to determine the time to QoL deterioration (TTD) in several different domains. TTD was significantly longer in the ^177^Lu-DOTATATE arm versus the control arm for global health status, physical functioning, role functioning, fatigue, pain, diarrhea, and disease related worries [14]. Additionally, as per patient reported symptom diaries that were completed in the NETTER-1 trial, patients experienced 3.11 fewer days of abdominal pain (*p* = 0.0007), 3.11 fewer days of diarrhea (*p* = 0.0017), and 1.98 fewer days of flushing (*p* = 0.0413) in the ^177^Lu-DOTATATE arm compared to the high-dose octreotide LAR group [15]. Despite a significant improvement in QoL and symptoms, 86% and 65% of patients in the ^177^Lu-DOTATATE group experienced side effects in the NETTER-1 and NETTER-2 trials, respectively [11,13].

Since ^177^Lu-DOTATATE approval, tumor flare reactions including increased pain and small bowel obstruction (SBO) have been reported [16,17]. These reactions are thought to be an inflammatory response to initial tumor injury, thus causing edema. When diseases are located in soft tissue deposits close to critical structures, the edema can cause subsequent organ or nerve dysfunction. Tumor flare reactions can result in decreased QoL or treatment delays and additional interventions for patients. These reactions have also been seen with external beam radiation to bone metastases, and given the similarities in mechanism, this is not surprising [18]. In these cases, dexamethasone has been shown to decrease the incidence of flare reactions [19,20].

A retrospective review of 22 patients treated with ^177^Lu-DOTATATE who were deemed to be high risk for SBO due to mesenteric and peritoneal disease burden found that 6% of patients experienced at least one episode of SBO within 3 months of treatment [17]. Other reactions included epigastric pain, cranial nerve dysfunction, and bone pain [16]. Another review found that in 12 patients treated with ^177^Lu-DOTATATE, 5 patients experienced a flare reaction including increased pain and SBO [16]. Both reviews report some success in treatment of the flare reaction with corticosteroids and the use of prophylactic corticosteroids to prevent flare reactions with future doses of ^177^Lu-DOTATATE. This has led to some centers utilizing corticosteroids in patients with NETs, with ^177^Lu-DOTATATE used to prevent tumor flare reactions in patients who are deemed high-risk.

There is evidence that use of corticosteroids can cause downregulation of somatostatin receptors [21]. There is currently no evidence that corticosteroids impact treatment outcomes in patients treated with ^177^Lu-DOTATATE; however, clinical trials recommend against their use. A single dose of corticosteroids was allowed in the case of refractory nausea and vomiting but was prohibited prior to treatment with ^177^Lu-DOTATATE and up to one hour after infusion [11,13].

Given that corticosteroids are used in practice to help prevent tumor flare reactions based on limited evidence, we aimed to assess if this practice was efficacious in our patient population.

## 2. Materials and Methods

In this retrospective, single institution study, we identified adult patients with NETs who were treated with ^177^Lu-DOTATATE between 1 October 2019 and 31 December 2024. Patients who were deemed high-risk for tumor flare reactions and received corticosteroids as prophylaxis were included. The high risk for flare reactions was defined by the treating physician and usually based on a high burden of disease, significant peritoneal or mesenteric disease, or disease involvement of critical structures.

This study was performed under the approval of the Institutional Review Board at Vanderbilt-Ingram Cancer Center, #231819 approved on 8 January 2024. Variables including demographics, diagnosis, treatment history, steroid dosing, and outcomes were collected within a RedCAP database. Descriptive statistics were calculated using Excel.

## 3. Results

Forty-six patients were identified as having received corticosteroid prophylaxis to prevent tumor flare reactions due to ^177^Lu-DOTATATE treatment (Table 1). Patients had a median age of 66, and 50% were female. The primary disease site was the small intestine (72%), followed by the pancreas (9%). The majority of patients had World Health Organization (WHO) grade 1 (41%) or WHO grade 2 (35%) diseases. While most patients (83%) received corticosteroids prior to the initiation of ^177^Lu-DOTATATE, 17% of patients received corticosteroids due to previously having a tumor flare after ^177^Lu-DOTATATE administration. All corticosteroid courses lasted for 7 days and started one to three days prior to treatment.

**Figure 1:**
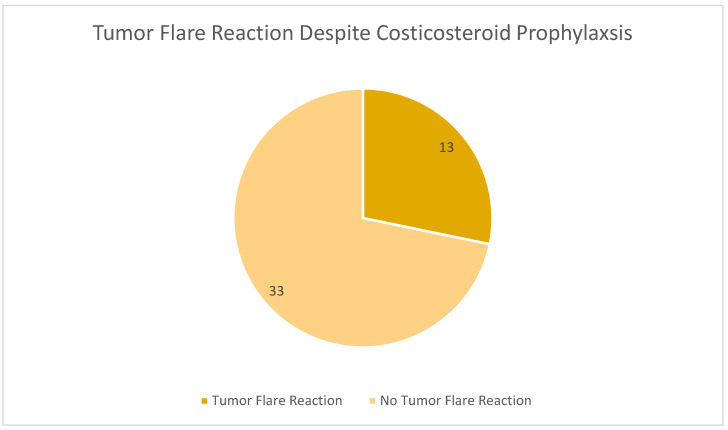
Number of patients who experienced a tumor flare reaction despite corticosteroid prophylaxis.

Despite corticosteroid prophylaxis, 28% of patients still experienced a tumor flare event (Figure 1), with three patients experiencing multiple tumor flare events. Small bowel obstruction occurred in 7% of patients and increased abdominal pain occurred in 22% of patients.

Adverse events (AEs) due to corticosteroids occurred in 28% of patients, with the most common AE being hyperglycemia and insomnia (Table 2). Other AEs experienced by patients included hypertension, diarrhea, gastrointestinal reflux disease (GERD), vivid dreams, and GI bleeding. Although most adverse events were grade 1 or 2 in nature, one patient experienced grade 3 GI bleeding, requiring hospitalization and intervention.

## 4. Discussion

In several small retrospective series, tumor flare reactions after ^177^Lu-DOTATATE administration occurred in 6% to 42% of patients [16,17]. These reactions are typically characterized by the worsening of pain or obstruction due to inflammatory responses secondary to tumor cell death. Subsequently, tumor flare reactions can result in treatment delays and increased healthcare resource utilization.

In our retrospective review of patients who received corticosteroid prophylaxis to prevent tumor flare reactions, 28% of patients experienced a tumor flare reaction with 22% of those patients experiencing increased abdominal pain and 7% of patients experiencing small bowel obstruction. In the NETTER-1 and NETTER-2 trials, increased abdominal pain occurred in 26% and 18% of patients, and small bowel obstruction occurred in 0% and 3% of patients, respectively [11,13]. Although a real-world patient population may include patients with poorer performance statuses or larger disease burdens, based on these numbers, it does not seem that corticosteroid prophylaxis improved the rates of tumor flare reactions and thus their role in this setting remains uncertain.

In our series, most patients tolerated corticosteroids well; however, we did see a significant number (28%) of patients experiencing AEs. The most common adverse events experienced by our patients were hyperglycemia, insomnia, edema, and leukocytosis. Although most AEs were grade 1 or 2, one patient experienced a gastrointestinal bleed requiring hospitalization and intervention which ultimately caused treatment delays for this patient and increased healthcare resource utilization. This reinforces the importance of weighing the risks and benefits associated with corticosteroid administration.

Two separate doses of corticosteroids were used in our patients. Fifteen patients received dexamethasone 4 mg twice a day for 7 days, starting 1 day prior to treatment. Thirty-one patients received dexamethasone 4 mg twice a day for 7 days, starting 3 days prior to treatment. There is sparse information available in previously reported data regarding the exact steroid dosages administered to patients; however, reported dosing ranged from 1 week of corticosteroid dosing to corticosteroid tapers over 2–3 weeks [16,17]. Given this limited information, there are no dosing recommendations included in the guidelines, and current doses used in practice are likely to vary; additionally, it is unclear what dose, if any, would provide meaningful benefit for patients.

In addition to questionable efficacy and safety concerns, corticosteroids may downregulate the SSTR, which would potentially impair the uptake of ^177^Lu-DOTATATE in patients [21]. This could subsequently lead to poor treatment outcomes or treatment failure. This is particularly concerning for patients with limited treatment options at baseline. Additionally, since clinical trials prohibited corticosteroid use prior to treatment with and up to one hour after ^177^Lu-DOTATATE, it is even more important to be prudent when considering the prescription of corticosteroids for these patients.

A limitation of this study is that it is single-center, retrospective, and involves non-randomized data with limited numbers that limit generalizability and can introduce bias. Furthermore, increased numbers would allow patient stratification based on tumor burden, prior treatments, and comorbidities to help identify patients who are at a higher risk of tumor flare reaction or corticosteroid-related complications.

A large, randomized clinical trial would be needed to definitively determine the efficacy and safety of corticosteroids in this setting, and to determine their potential impact on treatment outcomes. Additionally, the use of alternative options to decrease inflammation, such as NSAIDs (non-steroidal anti-inflammatory drugs) without compromising SSTR expression would be worthwhile.

## 5. Conclusions

Short-course corticosteroid prophylaxis to prevent tumor flare reactions in high-risk patients with NETs who had been treated with ^177^Lu-DOTATATE did not appear to decrease the incidence of tumor flare reactions when compared to previously reported studies. Randomized, placebo-controlled trials evaluating the use of corticosteroids to prevent tumor flare reactions in patients treated with ^177^Lu-DOTATATE are needed to fully elucidate the safety and efficacy of corticosteroid usage in this setting. Additionally, given the limited treatment options for patients with neuroendocrine tumors, the potential impact of corticosteroids on treatment outcomes in patients receiving PRRT need to be further explored.

## Figures and Tables

**Table 1:** Baseline demographics.

Characteristic	N = 46 ^1^ (%)	Characteristic	N = 46 ^1^ (%)
**Age**	66 (60.5, 71.5)	**Primary Disease Site**	
**Sex**		Small intestine	33 (71.7)
M	23 (50)	Pancreas	4 (8.7)
F	23 (50)	Lung	2 (4.4)
**Race**		Other	7 (15.2)
White	40 (87)	**Metastatic Disease Location**	
Black	5 (10.9)	Liver	34 (72)
Asian	1 (2.1)	Peritoneum/mesentery	13 (28)
**WHO Grade**		Bone	14 (30)
1	19 (41)	Lymph nodes	28 (61)
2	16 (35)	Lung	3 (7)
3	1 (2)	Other	13 (28)
Typical	1 (2)	**Previously had tumor flare reaction**	
Atypical	2 (4)	Yes	8 (17.4)
Unknown	7 (15)	No	38 (82.6)

^1^ Median (IQR); N (%).

**Table 2:** Adverse events due to corticosteroids.

Event	N = 46 ^1^
Any AE	13 (28)
Hyperglycemia	3 (7)
Insomnia	3 (7)
Edema	2 (4)
Leukocytosis	2 (4)
Other	5 (11)

^1^ Median (IQR); N (%).

## Data Availability

The data presented in this study are available on request from the corresponding author due to institutional ethical and legal reasons.
